# Synthesis and Characterization of Hydrogel-Based Hyaluronic Acid-Chitosan-*Allium sativum* Extract for Intraperitoneal Antiadhesion Application

**DOI:** 10.1155/2023/5172391

**Published:** 2023-03-03

**Authors:** Prihartini Widiyanti, Yolanda Citra Ayu Priskawati

**Affiliations:** ^1^Biomedical Engineering Study Program, Department of Physics, Faculty of Science and Technology, Universitas Airlangga, Surabaya, Indonesia; ^2^Institute of Tropical Disease, Universitas Airlangga, Surabaya, Indonesia

## Abstract

Peritoneal Adhesion is a severe case that frequently occurs in patients after laparotomy surgery. Adhesions are pathological attachment that usually appears between the omentum, intestine, and abdominal wall. Several barriers are made to prevent adhesions, including liquid barriers such as sodium hyaluronate and carboxymethyl cellulose (CMC) but are fast absorbed-time hydrogel. The solid barrier has weakness of difficulty in covering all parts of the wound surface. The study aims to synthesize degradable hydrogel from N,O-Carboxymethyl Chitosan (NOCC), Aldehyde-Hyaluronic Acid, and the addition of *Allium sativum* (garlic oil). The best sample with the concentration of A-HA/NOCC 30 : 10 g/ml was obtained. The composite hydrogel of NOCC/AHA/*Allium sativum* has susceptible antimicrobial properties. *In vitro* cytotoxicity assay showed that hydrogel is nontoxic. The degradation time is for two weeks. The in vivo evaluation in a mouse model with an abrasion defect side was done to identify the effectiveness of the NOCC/AHA/*A. sativum* as antiperitoneal adhesion. Seven days after surgery, the observation of adhesion was performed. Based on all assay results, it can be resumed that the NOCC/AHA/*A. sativum* hydrogel possibly acts as an innovation to prevent postoperative intraperitoneal adhesion.

## 1. Introduction

Intraperitoneal adhesions are mainly caused by surgery in the abdomen or pelvis. Causes of adhesion incidents intraperitoneal are about 67–93% formed after abdominal surgery. In a clinical study, patients with a previous laparotomy had a chance of developing about 70–90% intraperitoneal adhesions. Adhesions that form after surgery often cause chronic pain in the abdomen or pelvis, the difficulty of re-operative, infertility in women, intestinal obstruction, and even death. The onset of abdominal and pelvic pain is caused by the formation of adhesions resulting in nerve injury, tissue damage, and scar tissue formation [1]. Statistical data in Indonesia in 2004 recorded about 7,024 cases of patients with obstructive ileus who were hospitalized [2]. The percentage of subjects with obstruction of the small intestine is 73.9%, while in significant bowel obstruction, it is 26.1% [3]. Intraperitoneal adhesions are very dangerous and almost always occur after major intraperitoneal surgery. Re-operative procedures to remove adhesion will tend to be more difficult, risky, and dangerous. This re-operative will prolong the operation time, anesthesia, and recovery time and cause risks such as blood loss and visceral damage, including bladder injury, intracutaneous fistula, and bowel damage [1].

The development of intraperitoneal adhesions has been studied broadly. But to date, there is no definite approach to impede its arrangement because controversy over the efficacy of preventive agents still exists [4]. Currently, the most potent way to prohibit adhesion formation is to utilize physical hurdle materials that separate from the network, thereby diminishing the probability of contiguous connection [5]. However, an exemplary surgical technique is not effective enough to eliminate the formation of intraperitoneal adhesions [6]. Many studies have been carried out to prevent current adhesion formation. Some of them are chemical drugs such as corticosteroids, anticoagulants, antibiotics, fibrinolytic agents, hormones, anti-inflammation, and anticoagulants, including aspirin, dexamethasone, and heparin. Barrier liquids such as crystalloids (NaCl and Ringer's lactate) and polymer solutions (N,O-carboxymethyl Chitosan (NOCC), sodium hyaluronate, and carboxymethyl cellulose (CMC)), although used in large quantities but too fast absorbed [7]. Solid barriers are still challenging to apply because they are difficult to cover all parts of the wound surface and often stick on the surgeon's glove when inserted into the body [8].

In the process of treating the problem of the emergence of intraperitoneal adhesions, anti-adhesion barrier hydrogel innovation was found. In recent years, world researchers have developed hydrogels as a physical barrier solution adhesion. The use of hydrogels for anti-adhesion inhibitor solutions has attracted much interest and attention among researchers. This hydrogel can be applied with injectable, spray, or laparoscopic methods [9]. Injectable Hydrogels are an attractive tissue application because they have a kind of structure with the extracellular matrix and are minimally invasive applications. Gelation time dramatically affects the application of injectables because the long setting time is necessary for hydrogel can cover the wound area perfectly. The cells covered by hydrogel will stimulate the release or production of ECM (maximum extracellular) to restore damaged tissue. Then this hydrogel can maintain cells in the affected tissue area damaged to carry out cell growth or recovery, metabolism, and new ECM synthesis. Hydrogels are easily degraded when cells secrete ECM, so the hydrogel needs to be modified or composite with other polymers [5]. Hydrogels have the potency to impersonate extracellular matrix (ECM), which is helpful in tissue regeneration for wound healing. The hydrogel can also cover all parts of the infected wound area to isolate the wound surface from other contiguous tissues or organs to prevent the formation of adhesive tissue or intraperitoneal adhesions after surgery within a certain period [10]. According to Li's research et al. [11], the ideal anti-adhesion material should have the following characteristics such are: have a degradation period of 7 days post-operatively, biocompatible, safe, non-inflammatory, non-immunogenic, last long critical re-mesothelialization phase, could adhere without sutures, could function in the presence of blood, and keep the intraperitoneal surfaces isolate to avoid the connection between destructed serous surfaces for 5–7 days for epithelialization intraperitoneal.

In a previous study performed in 2014 by Li et al. which is to create an innovation of -based post-operative adhesion prevention injectable hydrogel, which is biodegradable by composite natural polysaccharides, namely N,O-carboxymethyl Chitosan (NOCC) and aldehyde hyaluronic acid (A-HA) with a concentration ratio of A-HA: NOCC of 30: 20 mg/mL. The characterization of this hydrogel biocomposite shows that the *in vitro* cell viability test showed that the hydrogel was not toxic at a concentration ratio of A-HA: NOCC of 30 mg/ml: 20 mg/ml. It is also known that the morphology test with SEM shows that hydrogel has good porosity and interconnection so that it supports cell growth and proliferation in areas encapsulated by hydrogel and can become a hurdle to the connection of fibroblasts to the surface intraperitoneal. However, in this study, the degradation period of up to 14 days. Meanwhile, the ideal anti-adhesion physical barrier standard must have a seven-daypost-operatively degradation period. It is known that intraperitoneal. The parietal mesothelia regenerate in about 5–6 days, and the visceral period lasts 5–8 days [11]. The hydrogel inhibits fibroblast invasion during the critical period of mesothelia regeneration to prevent adhesions. If the hydrogel is degraded more than seven days after surgery, the residual material from the hydrogel in the intraperitoneal can affect the re-mesothelialization process [12]. So that system in the body will elicit a response that considers hydrogel as a foreign object and must be destroyed so that it can jeopardize the healing process of the intraperitoneal membrane [13].

Hyaluronic acid is a polyanionic polysaccharide that is a major component of the extracellular matrix [14] which is biodegradable, biocompatible, hydrophobic, non-immunogenic, and has high viscosity (viscosity) properties. It is reported that hyaluronic acid can also raise the proliferation of intraperitoneal mesothelial cells. However, it has the weakness of too fast degradation and strength-poor mechanics [11]. Therefore, Chitosan was added to improve the nature of the degradation that is too fast. Chitosan is a polysaccharide that is commonly found in nature [15]. Chitosan is also biocompatible because glucosamine from the extracellular metric (ECM) is similar to chitosan structure, is biodegradable and easy to composite with other ingredients, and has hemostatic properties that can help prevent post-operative bleeding and has antimicrobial properties [16]. The Alliaceae family's, garlic (*Allium sativum Linn.*) is a plant that grows to 30–60 cm. This species has several uses: antioxidant, insecticidal, antinociceptive, antitrypanosomal, and antimicrobial. In Sahbaz et al. research [17], it has been proven that Allium sativum has antibacterial, anti-inflammatory, antithrombotic, fibrinolytic, antioxidant, and curing properties to prevent the appearance of post-operative peritoneal adhesions. Allium sativum can also kill *Staphylococcus aureus*, *Streptococcus viridans*, and *Escherichia coli*. Based on this background, a hydrogel physical barrier was created as a degradable adhesion barrier by composite hyaluronic acid–Chitosan, modification of the concentration of Chitosan was carried out in hyaluronic acid to determine the best concentration ratio in hydrogel biocomposite so that it can be degraded right after the healing period finished.

## 2. Materials and Methods

The materials utilized in this research were Hyaluronic acid (AH), Chitosan, Sodium metaperiodate (NaIO_4_), Isopropyl Alcohol (IPA), NaOH, Monochloroacetic Acid, Methanol, Alcohol, Distilled water, Ethylene Glycol, Normal Saline (NS), and Phosphate Buffered Saline (PBS), Garlic oil.

### 2.1. Synthesis of Aldehyde-Hyaluronic Acid A-HA

Hyaluronic Acid is arranged by blending powder with distilled water to produce a transparent and viscous mixture. Then the sodium metaperiodate (NaIO_4_) was added in dark conditions to stimulate a periodic oxidation reaction to gain an aldehyde group. Finally, ethylene glycol was added to block periodic oxidation reactions and produce a clear and thick solution. After that, the mixture was dried with a freeze-dryer.

### 2.2. Synthesis NOCC

NOCC is prepared by mixing chitosan powder with isopropyl alcohol to yield a yellowish turbid solution. NaOH, as pH control, was added to the solution. Then the addition of acid monochloroasetate dripped slowly until it formed carboxymethyl chitosan. It was then stirred at 60°C for about 3 hours until homogeneously. The chitosan solution supernatant was filtered using filter paper. The purification and washing step of Chitosan using methanol and 70% alcohol. It showed a very thick yellow solution. Chitosan was dried with a freeze-dryer.

- Control sample with a ratio of AH: chitosan of 30 : 0 mg/ml
- Sample variation of AH: chitosan ratio of 30 : 10 mg/ml
- Sample variation of AH: chitosan ratio of 30 : 20 mg/ml
- Sample variation of AH: chitosan ratio of 30 : 30 mg/ml

### 2.3. Characterization

The cross-linking between the AHA with NOCC to form NOCC/AHA was performed at concentrations of 30 : 0, 30 : 10, 30 : 20, and 30 : 30 mg/ml with a volume ratio of 1 : 1.

### 2.4. Fourier Transform Infra Red (FTIR)

Fourier Transform Infrared Spectroscopy (FTIR) is an analytical technique used to identify organic, polymeric, and in limited cases, in inorganic materials. FTIR test observes the functional groups of compounds, and in this study using the FTIR IRTracer-100 Shimadzu. First, hydrogel sample based on hyaluronic acid-chitosan to be mixed with KBr powder, put on a platinum pan and will be penetrated by infrared rays. The *x*-axis is referred to wave number of compound, and *y* axis is referred to the percentage transmittance. The functional group graph analysis is performed by correlate transmittance band at infrared spectrum and handling with the compound spectrum comparison [11].

### 2.5. Swelling Test

The swelling test was carried out to determine the sample's capability to absorb liquid. The calculation of the swelling ratio was performed by the ratio of the wet weight of the sample and the weight before immersion in PBS solution for 24 hours at 37°C [18]. First, blotting paper was used to dry the hydrogel surface, and was weighed before and after immersion. Then, the swelling ratio percentage was calculated using the equation:

(1) $$\begin{array}{c} \%SR=\frac{Wwet-Wdry}{Wdry}x100\%\text{,} \end{array}$$

%SR = Percentage of swelling ratio. *W*wet = Weight wet (weight subsequent sample dyeing). *W*dry = weight dry (initial weight before drying).

### 2.6. *In Vitro* Degradation of Hydrogels

*In vitro* degradation of hydrogels test determines the ability of the sample to survive when implanted into the body, so it is necessary to carry out a degradation test. This test was carried out by immersing the sample in a Phosphate Buffer Saline (PBS) solution with a neutral pH of 7.4, which was incubated at 37°C for 7, 14, and 21 days [18]. Next, the weight loss of the sample was observed. Finally, the percentage of sample weight is calculated using the following equation:

(2) $$\begin{array}{c} ∆W\%=\frac{W0-Wt}{W0}\times 100\text{,} \end{array}$$

Δ*W* = percentage of sample weight lost. *W*_0_ = Initial weight before sample dyeing. *W*_*t*_ = Weight subsequent sample dyeing.

### 2.7. Antibacterial Test

This test was conducted to determine the antibacterial activity of the chitosan/BKP membrane. The method used is disc diffusion in a petri dish. Bacterial cultures of *Escherichia coli* and *Staphylococcus aureus* were injected into agar media. After solidification to coat the agar medium, chitosan-BKP membranes (15 mm diameter) with different concentrations (0, 9, 10, and 11% wt) were placed on the surface of the agar media. The layers were incubated at 37°C for 18–24 hours. The zone of inhibition on the growth of *Escherichia coli* and *Staphylococcus aureus* bacteria can be seen by the presence or absence of a clear area formed around the sample [19].

### 2.8. *In Vitro* Test Cytocompatibility

*In vitro* cytocompatibility test was carried out using cell culture to explore a material's lethal effect directly. By doing this test, possible to obtain some information on the probability of survival cells. MTT Assay method using 96 microplates and based on the capability of a living cell to diminish MTT salts. Hepatocyte cell cultures were incubated for 48 hours on Eagle's medium. Then the results were obtained through the ELISA reader. If the number of living cells is more than 50%, the test material can be said to be safe or non-toxic [20]. This test will produce the color intensity of the formazan product, which is purplish-blue. The darker the color produced, the higher the absorbance value, which means more cells are still alive. The percentage of surviving cells can be calculated using equation (11):

(3) $$\begin{array}{c} \%Living\;cells=\frac{absorbance\;sampel}{\sum absorbance\;cell\;control}x100\text{.} \end{array}$$

### 2.9. *In Vivo*Anti-Adhesion Evaluation of NOCC/AHA/*A. sativum* Hydrogel

The *in-vivo* test is preceded by the approval of animal ethics and research protocol from the Ethics Committee of the Faculty of Veterinary Medicine, University of Airlangga no. 2.KE.066.04.2018. This study uses a species of rat (*Rattus norvegicus*) female weighing about 200 gr–250 gr, aged 2-3 months. First, the surgeon made the wound by performing a vertical incision in the peritoneum. There were three control groups with a ratio of AH : Chitosan of 30 : 10 mg/ml (the best composition due to another assay result), negative control with group AH : Chitosan ratio of 30 : 0 mg/ml, and positive control with a normal saline group. Then, seven days after surgery, the observation of adhesion (gross anatomy observation/clinical manifestation) was performed.

## 3. Results and Discussion

### 3.1. Characterization

#### 3.1.1. Fourier Transform Infrared Spectroscopy

**Figure 1:**
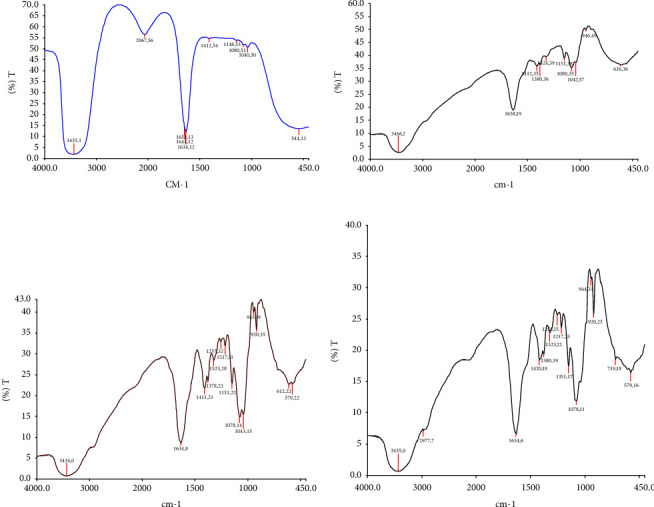
FTIR spectra of hyaluronic acid (HA): chitosan hydrogel with varying composition HA: chitosan sample (a) (30 : 0 mg/ml), sample (b) (30 : 10 mg/ml), sample (c) (30 : 20 mg/ml), sample (d) (30 : 30 mg/ml).

This research produced a sample of hydrogel based on hyaluronic acid (HA)-chitosan-*A. sativum*. In the hydrogel formation process, the freeze-dried samples were dissolved in normal saline and divided into four concentration groups: A, B, C, and D with varying concentrations of HA: Chitosan 30 : 0, 30 : 10, 30 : 20, 30 : 30 mg/ml (as seen in Figure 1).

To determine the cross-link between the two materials, AHA and NOCC, indicated by a sharp absorption peak at a wave number of about 1638 cm^−1^ stretching C=N, indicates a Schiff base relationship (-CH=N-). This cross-link appears between the amino group (NH_2_) and the aldehyde group (CHO). In addition, *Allium sativum* produces amide I amide III group, a component of protein at wave numbers 1630 cm^−1^ and 1250 cm^−1^.

### 3.2. Equilibrium Swelling

**Figure 2:**
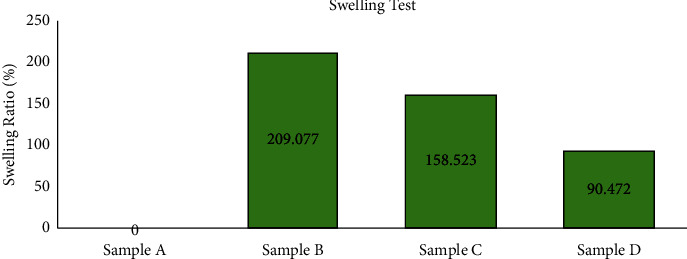
The swelling test results of hyaluronic acid (HA): chitosan hydrogel with varying composition HA: chitosan sample A (30 : 0 mg/ml), sample B (30 : 10 mg/ml), sample C (30 : 20 mg/ml), sample D (30 : 30 mg/ml).

Subsequently, a swelling test was performed on each hydrogel sample to determine the sample's capability to absorb fluids in the body. The swelling test results are shown in Figure 2. Sample A did not swell because sample A was in fluid form, so the sample was directly hydrolyzed with PBS liquid. However, samples B, C, and D experienced swelling, each producing a percentage of 209.077%, 158.523%, and 90.472%. The standard effective swelling ratio of hydrogel for intraperitoneal adhesion application is 123–225% [21]. Therefore, it can be concluded that the higher the chitosan concentration from the hydrogel, the more amino groups cross-linked with the HA aldehyde group, causing a slight reaction residue and low swelling ability.

Conversely, if the chitosan concentration is lower, the absorption capacity will be higher. It is known that the concentration of Chitosan, the higher the hydrogel, the more amino groups bond so that more cross-links occur, causing the distance between The molecules in the hydrogel is getting closer and closer together. These results showed that the smaller the pore size, the harder it is for water to diffuse into the hydrogel and the swelling ability is low [11].

### 3.3. *In Vitro* Degradation

The degradation test was carried out with variations on days 3, 7, and 14. Sample A showed 100% degradation on the first day due to the liquid form of the sample. Meanwhile, samples B, C, and D were degraded by 90.5%, 87%, and 83.5%. The test results showed that the higher the concentration of Chitosan, the longer the degradation rate. This is because the greater the concentration of Chitosan, the greater the cross-link between the aldehyde and amine groups. This can produce the a greater sample density, and the porosity will be low. If there is low porosity, PBS will be more burdensome to enter hydrogel and diminish the degradation rate. The endurance of Chitosan to face degradation is influenced by the distribution of acetamide groups in the chitosan molecule. This happened due to differences in deacetylation status, affecting the chitosan solution viscosity by improving the inter- or intra- molecular repulsion forces. However, it is inconceivable to predict the biodegradation rate of Chitosan using the degree of acetylation alone [22]. The molecular weight of Chitosan might modify from 300 to over 1000 kD, which alters many of its characteristics, such as viscosity. Thus, the membranes will be more viscous if Chitosan have great molecular weight. Based on its high molecular weight and direct nonbranched structure, Chitosan is a forceful viscosity-constructing agent in acid mediums. It operates as a pseudoplastic material, where viscosity leans on disturbance. There is a linear corporation between molecular weight and degree of acetylation [23].

### 3.4. Antibacterial Test

**Figure 3:**
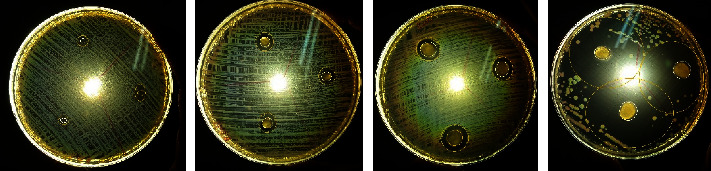
Antibacterial testing results of of hyaluronic acid (HA): chitosan hydrogel with varying composition HA: chitosan sample A (30 : 0 mg/ml), sample B (30 : 10 mg/ml), sample C (30 : 20 mg/ml), sample D (30 : 30 mg/ml).

The antibacterial testing results of Hyaluronic Acid (HA): Chitosan Hydrogel with varying composition HA: Chitosan sample A (30 : 0 mg/ml), sample B (30 : 10 mg/ml), sample C (30 : 20 mg/ml), sample D (30 : 30 mg/ml) can be seen in Figure 3.

Antibacterial testing was carried out on all samples using *Staphylococcus aureus* and *Escherichia coli* bacteria to set the resistance of the sample to bacteria. This antibacterial test was focused on the efficiency of chitosan variations and the addition of *Allium sativum*, which has advantages in inhibiting bacterial growth. From the test, the data is generated as in Table 1. Sample A shows resistance. There are microorganisms classified as sensitive. Meanwhile, samples B, C, and D were classified as very sensitive to microorganisms because they have. Besides the effect of Chitosan, *Allium sativum* oil, which has an antibacterial effect, can also strengthen the potency of the hydrogel as an antibacterial anti-adhesion agent for intraperitoneal organs [24]. Therefore, a higher concentration of Chitosan will have better antibacterial activity and vice versa. The mechanism of the antibacterial property of Chitosan is by binding to the negatively charged bacterial cell wall generating interruption of the cell, then improving the membrane permeability, then by connection to DNA generating reticence of DNA replication and followed by cell death [25]. Another feasible mechanism is that Chitosan plays a chelating agent that selectively binds to trace metal elements generating toxin production and constraining microbial growth [26]. The polycationic structure of Chitosan is a prerequisite for antibacterial activity. If environmental pH is below the pKa of Chitosan and its derivatives, electrostatic interaction between the polycationic structure and the predominantly anionic components of the microorganisms' surface (such as Gram-negative lipopolysaccharide and cell surface proteins) plays an essential role in antibacterial action [27].

### 3.5. *In Vitro* Test Cytocompatibility

**Figure 4:**
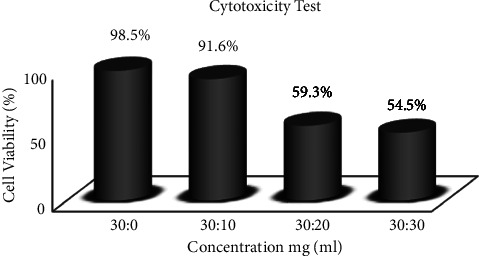
Cytotoxicity test results.

The Cytotoxicity test results (as seen in Figure 4) showed that each sample's cell viability was 98.5%, 91.6%, 59.3%, and 54.5%. This indicates that samples A, B, C, and D are non-toxic because they have more than 50% cell life sustainability [21].

In vivo testing was carried out using Wistar rats (*Rattus norvegicus*) 2-3 months old, weighing 200 g–300 g. The best sample was obtained with a concentration of HA: Chitosan of 30 : 10 mg/ml. The treatment group was divided into three groups, as shown in Table 2.

**Figure 5:**
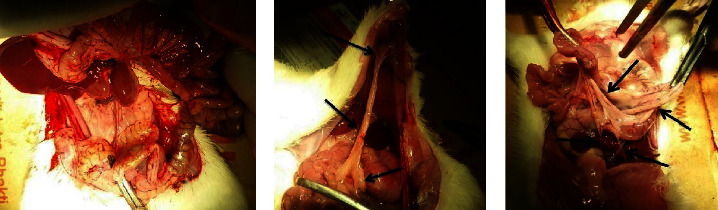
(a) No adhesion, (b) thin adhesion, (c) thick adhesion.

First, the surgeon performed a sidewall defect-cecum abrasion model. After seven days, a second surgery was performed to separate adhesions by a proper dissection. Then the separated abdominal wall and cecal surface were re-rub with sterile gauze. NOCC-AHA-*A. sativum* hydrogel was utilized to coat both the wounded and the unwounded region. The temperature of the abdominal cavity can act as a booster for the formation of NOCC-AHA hydrogel. Therefore, the reliable hydrogel was constructed in situ. Two weeks later, the rats were sacrificed to appraise the existence of adhesion. In the control (−) group, all testing animals showed a score of 5 adhesion, which indicated the successful formation of the repeated-wounded adhesion model (Figure 5(c)). Normal saline/control (+) group showed a score of 4 adhesion, which indicated poor anti-adhesion effectiveness and had no significant disparity compared with control (−) group. It can be seen in Figure 5 that no adhesion was found in the group given A-HA/NOCC/*A. sativum* hydrogel. Meanwhile, in the positive control group, adhesions were found between the organs and the abdominal wall, likewise in the negative control group, which resulted in more adhesion between the abdominal organs of experimental animals. Therefore, it can be concluded that hydrogel A-HA/NOCC/*A. sativum* is effective in preventing the formation of post-operative adhesions of the abdomen.

## 4. Conclusions

AHA/NOCC/*A. sativum* hydrogel showed good properties for adhesion prevention due to swelling properties, degradability, biocompatibility, antibacterial property, and its ability to prevent adhesion formation (*in vivo*). Therefore, the A-HA/NOCC/*A. sativum* hydrogel can be considered as candidate for intraperitoneal anti-adhesion.

## Figures and Tables

**Table 1:** Antibacterial testing results.

Sample	*Staphylococcus aureus*		*Escherichia coli*	
	Zona (mm)	Resistance	Zona (mm)	Resistance
A	20	Sensitive	5	Sensitive
B	25	Very sensitive	14	Very sensitive
C	30	Very sensitive	20	Very sensitive
D	65	Very sensitive	45	Very sensitive

**Table 2:** *In vivo* test of adhesion.

Group	Condition
Control (−)	Without hydrogel
Control (+)	Normal saline
Hydrogel (HA: chitosan 30 : 10 mg/ml)	With hydrogel

## Data Availability

The data supporting the findings of this study are available within the article.
